# AI-based prediction of heart failure progression in persistent atrial fibrillation using wearable electrocardiography: a brief research report

**DOI:** 10.3389/fcvm.2025.1748673

**Published:** 2026-02-05

**Authors:** Chae-Bin Song, Yun Kwan Kim, YouMi Hwang

**Affiliations:** 1 Department of Technology Development, Seers Technology Company Ltd., Pyeongtaek, Republic of Korea; 2 Department of Brain and Cognitive Engineering, Korea University, Seoul, Republic of Korea; 3 Department of Cardiology, College of Medicine, St. Vincent’s Hospital, The Catholic University of Korea, Suwon, Republic of Korea; 4 College of Medicine, Catholic Research Institute for Intractable Cardiovascular Disease (CRID), The Catholic University of Korea, Seoul, Republic of Korea

**Keywords:** artificial intelligence, atrial fibrillation, digital health, electrocardiography, heart failure, machine learning, wearable device

## Abstract

**Background:**

Persistent atrial fibrillation (AF) frequently coexists with heart failure (HF), yet HF monitoring remains limited by the need for repeated blood-based biomarkers such as N-terminal pro-brain natriuretic peptide (NT-proBNP). Advances in wearable electrocardiography (ECG) and artificial intelligence (AI) now allow continuous extraction of digital physiologic signatures that may reflect hemodynamic stress.

**Objective:**

To evaluate the feasibility of predicting HF progression using wearable ECG–derived features in patients with persistent AF.

**Methods:**

Fifty patients with persistent AF underwent 3–7 days of single-lead ECG monitoring. Heart rate variability (HRV) and RR-interval features from 30 min windows were combined with baseline clinical metrics. A context-aware deep learning model using long short-term memory (LSTM) and attention mechanisms was trained to predict 6–12-month NT-proBNP changes. Model performance was assessed using root mean squared error (RMSE), mean absolute error (MAE), and the accuracy of directional NT-proBNP change.

**Results:**

The best performance was achieved when clinical metrics, RR features, and long-term HRV summaries were combined (RMSE 1,667.04; MAE 950.52). Directional classification of NT-proBNP trajectories achieved an accuracy of 0.82. ECG-only models performed comparably to multimodal models.

**Conclusion:**

Wearable ECG–based AI modeling is feasible for predicting trends in HF biomarkers in persistent AF. These results provide early evidence that ECG-derived digital biomarkers may offer a scalable, non-invasive approach for longitudinal HF monitoring.

## Introduction

Atrial fibrillation (AF) and heart failure (HF) share complex bidirectional interactions that accelerate disease progression and worsen outcomes (1, 2). N-terminal pro-brain natriuretic peptide (NT-proBNP) reflects ventricular wall stress but requires in-hospital blood sampling, limiting its use for longitudinal monitoring (3, 4). In persistent AF, beat-to-beat irregularity and autonomic fluctuations further complicate the interpretation of conventional biomarkers.

Wearable ECG devices enable continuous rhythm monitoring and extraction of digital biomarkers such as heart rate variability (HRV) and RR-interval dynamics (5–7). Concurrent advances in artificial intelligence (AI) have shown that ECG-based algorithms can detect subtle physiologic signatures associated with ventricular dysfunction and HF events (8–10). In parallel, clinical biomarkers remain essential for assessing hemodynamic status. Longitudinal NT-proBNP trends may better reflect chronic hemodynamic stress than single measurements, especially in persistent AF.

This study aimed to evaluate the feasibility of predicting HF progression using an AI model trained on wearable ECG-derived features in patients with persistent AF. We hypothesized that ECG-based digital biomarkers could serve as surrogates for conventional laboratory indices such as NT-proBNP and facilitate personalized HF monitoring.

## Methods

### Study population

This prospective observational study enrolled 50 patients with persistent AF who underwent wearable ECG monitoring between 2023 and 2024 at St. Vincent's Hospital, The Catholic University of Korea. Adults aged 20 years and older were eligible. Exclusion criteria included acute decompensated HF, pacemaker rhythm, incomplete follow-up data, excessive motion artifact (>20% unusable signal), and <48 h of analyzable ECG. Follow-up NT-proBNP measurements were obtained during routine clinical visits, resulting in a sampling interval of 6–12 months. All participants provided written informed consent. The study was approved by the institutional review board (VC23OISI0082) and conducted in accordance with the principles outlined in the Declaration of Helsinki.

### Wearable ECG monitoring and feature extraction

Participants wore a single-lead wearable ECG device (MobiCARE, Seers Technology Co., Korea) continuously for 3 to 7 days. The ECG was segmented into 30 min windows to derive sequential HRV features (HRV30 min). Long-term HRV summaries and RR-interval–based features (HRV72 h and RR) were calculated from the entire recording period. Baseline clinical metrics (CM) were extracted from electronic medical records. Feature distributions were assessed using Q–Q plots and the Shapiro–Wilk test. All features were standardized using z-score scaling and Yeo–Johnson transformation. A 30 min window length was used to balance temporal resolution and noise reduction in persistent AF. Feature groups are summarized in Table 1.

**Table 1:** Definition of feature groups used in the analysis.

Feature group	Description	Included features
CM	Clinical variables collected from medical records	Age, BMI, HBP, DM, CVA, HFpEF, AAD, NOAC, Antiplatelet, BB, CCB, Hb, platelet, creatinine, uric acid, e/e′, EF(TTE), TR(TTE), baseline BNP
RR	RR interval-related features extracted from 72 h ECG summary statistics	minRR, maxRR, avgRR
HRV_72 h_	HRV features derived from 72 h ECG summary statics	minHR, maxHR, avgHR
HRV_30 min_	HRV features computed from 30 min ECG segments to capture sequential physiological information	meanNN, SDNN, RMSSD, NN50, pNN50, LF, HF, LF/HF, TP

AAD, antiarrhythmic drug; ACEi, angiotensin-converting enzyme inhibitor; AF, atrial fibrillation; ARB, angiotensin II receptor blocker; BB, beta-blocker; BMI, body-mass index; BNP/NT-proBNP, B-type natriuretic peptide/N-terminal pro-B-type natriuretic peptide; CAD, coronary artery disease; CCB, calcium-channel blocker; Cr, serum creatinine; CT Vol, computed-tomography-derived cardiac volume; CVA, cerebrovascular accident; DM, diabetes mellitus; e/e′, early mitral inflow velocity to mitral annular early diastolic velocity ratio; EF, ejection fraction; Hb, hemoglobin; HBP, hypertension; HF, heart failure; HFpEF, heart failure with preserved ejection fraction; HR, heart rate; HRV, heart-rate variability; LF/HF, low-frequency/high-frequency spectral power of HRV; NOAC, non–vitamin K oral anticoagulant; RMSSD, root mean square of successive differences; SDNN, standard deviation of normal-to-normal RR intervals; TP, total power (HRV index); TR Vmax, tricuspid regurgitation maximal velocity.

ECG preprocessing was performed using device-generated R-peak annotations together with noise-labeled intervals. R-peaks occurring within noise-marked segments were removed, and no additional ectopic-beat filtering was applied, consistent with the physiology of persistent AF. For each 30 min window, RR intervals were computed from successive R-peak timestamps. Windows with fewer than two valid RR intervals were treated as invalid, and all HRV metrics were coded as −1. Time-domain HRV metrics were computed directly from the cleaned RR series. At the same time, frequency-domain features were derived by converting RR intervals to seconds, applying linear interpolation to obtain an evenly sampled signal, and estimating power spectra using Welch's method. Windows with insufficient RR data for spectral estimation likewise had frequency-domain HRV metrics assigned a value of −1.

Although conventional short-term HRV measures have limited physiological interpretability in persistent AF because beat-to-beat irregularity obscures sinus-node–mediated autonomic modulation, a prior study has shown that long-window HRV and RR-variability–derived metrics can still reflect global autonomic tone and hemodynamic burden in AF. Based on this evidence, we used 30 min aggregated windows as surrogate markers of autonomic dynamics, as longer windows reduce stochastic irregularity and capture lower-frequency physiologic patterns relevant to AF-related remodeling (11).

To ensure signal reliability, artifact-related noise was mitigated by excluding recordings with more than 20% unusable segments, removing physiologically implausible RR intervals, and applying window-level aggregation, which collectively improves robustness in AF-derived HRV estimation.

### Model development

A hybrid deep learning model was developed to integrate time-varying ECG-derived features with static clinical information. For each patient, HRV features, RR-interval dynamics, and interaction terms were aggregated into a multivariate time series that reflects autonomic and beat-to-beat variability over the monitoring period. Clinical metrics, which are inherently non-temporal, were replicated across all 30 min windows and concatenated with the sequential features to create a unified model input. This allowed the network to learn temporal autonomic patterns while simultaneously leveraging stable patient-specific characteristics. Baseline NT-proBNP and meanRMSSD were also incorporated as context vectors within the attention mechanism to modulate temporal weighting in a patient-dependent manner.

The model architecture consisted of a three-layer long short-term memory (LSTM) network with 64, 32, and 16 hidden units, followed by a context-aware attention module and a two-layer regression head. This design enabled the model to capture both short-term fluctuations and longer-range patterns in autonomic variability. Training was performed using the Adam optimizer (learning rate 0.001), a batch size of 8, and mean squared error as the loss function, with early stopping applied based on training performance (patience = 10). To minimize overfitting in this small-sample setting, several regularization strategies were used, including L2 weight decay (0.001) and dropout (0.5).

Additional details regarding the model architecture, attention mechanisms, input construction, and statistical feature analysis are provided in the Supplementary Methods.

### Evaluation

Model performance was assessed using leave-one-out cross-validation (LOOCV) at the patient level, ensuring that no information from the held-out patient was used in model training. For each iteration, the model generated a continuous prediction of the change in NT-proBNP, which was subsequently categorized as an increase or a decrease. The primary endpoint was the predicted change in NT-proBNP. Performance metrics included root mean squared error (RMSE), mean absolute error (MAE), and directional accuracy.

## Results

### Study population

Among the 50 patients included in the analysis, the median age was 70.0 [60.25–78.75] years, and 56% were male. Baseline clinical characteristics are shown in Table 2.

**Table 2:** Baseline demographic, clinical, and echocardiographic characteristics of the study population (*n* = 50).

Variable	Value
Male, *n* (%)	28 (56.0%)
Age [median (IQR)]	70.0 [60.25–78.75]
BMI [median (IQR)]	24.76 [22.37–26.27]
HBP, *n* (%)	38 (76.0%)
DM, *n* (%)	15 (30.0%)
CVA, *n* (%)	3 (6.0%)
HFpEF, *n* (%)	50 (100.0%)
CKD, *n* (%)	2 (4.0%)
NYHA_class	I: 6 (12.0%); II: 32 (64.0%); III: 12 (24.0%)
anriarrhythmic drug, *n* (%)	18 (36.0%; Amiodarone 17, Sotalol 1)
Warfarin	0 (0%)
NOAC, *n* (%)	48 (96.0%)
Antiplatelet, *n* (%)	3 (6.0%)
ARB/ACEi, *n* (%)	30 (60.0%)
Beta blocker, *n* (%)	36 (72.0%)
calcium channel blocker, *n* (%)	2 (4.0%)
Creatinine (mg/dL) [median (IQR)]	1.0 [0.70–1.10]
BNP (pg/mL) [median (IQR)]	1,344.0 [708.0–3,527.5]
LAvolume (mL) [median (IQR)]	168.8 [167.1–191.1]
e/e' [median (IQR)]	11.07 [8.2–15.6]
LVEF(%) [median (IQR)]	59.8 [54.8–62.0]

ACEi, angiotensin-converting enzyme inhibitor; ARB, angiotensin II receptor blocker; BMI, body-mass index; BNP, B-type natriuretic peptide; CVA, cerebrovascular accident; DM, diabetes mellitus; e/e′, early mitral inflow velocity to mitral annular early diastolic velocity ratio; EF, ejection fraction; HBP, hypertension; HFmrEF, heart failure with mildly reduced ejection fraction; HFpEF, heart failure with preserved ejection fraction; HFrEF, heart failure with reduced ejection fractionNOAC, non–vitamin K oral anticoagulant.

### Model performance

**Figure 1:**
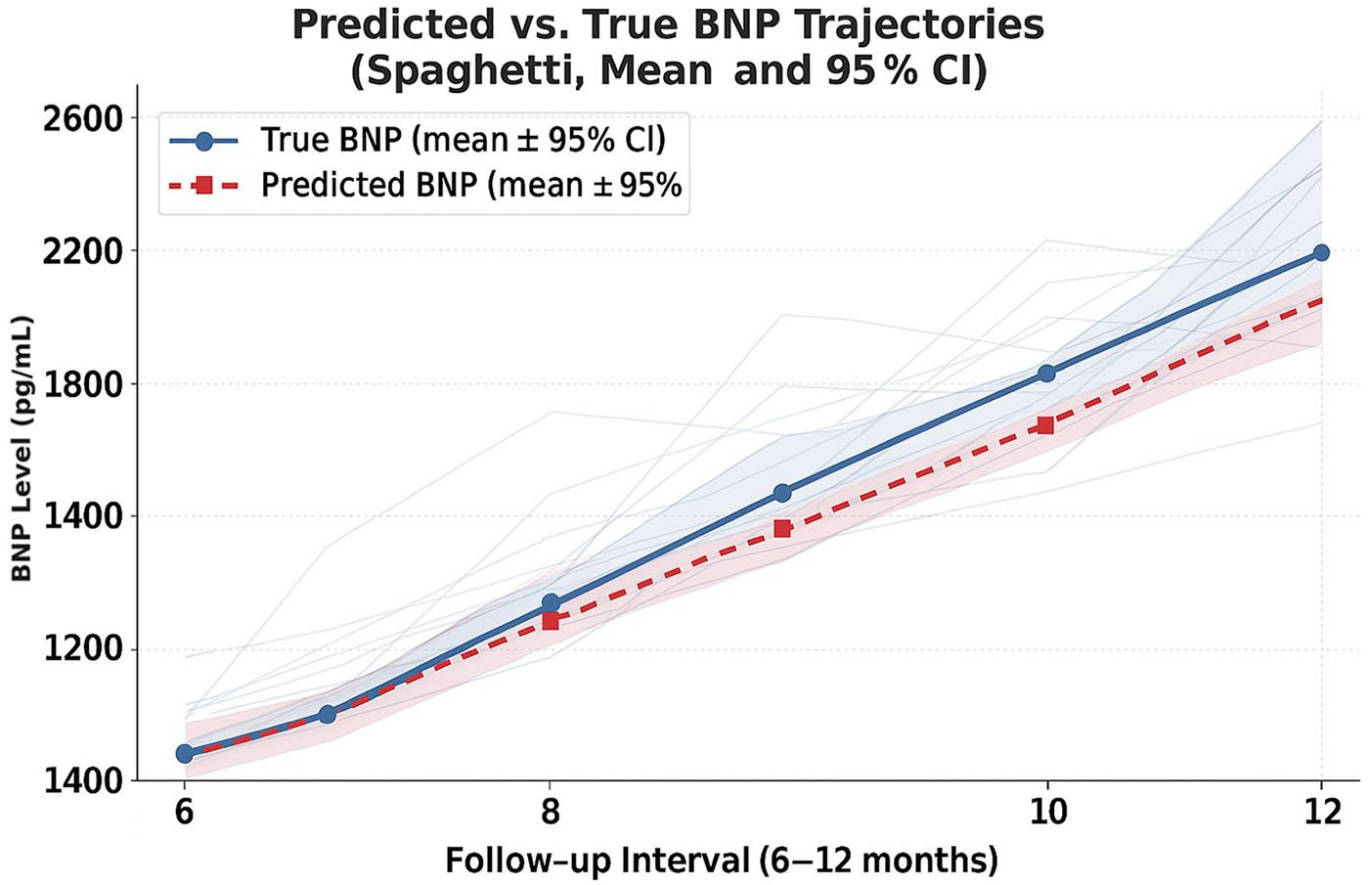
Predicted vs. observed NT-proBNP trajectories over the 6–12-month follow-up period (median 8.1 months, IQR 3.5 months), showing mean ± 95% CI and individual patient trajectories (spaghetti lines). Individual NT-proBNP trajectories (gray spaghetti lines) for all patients, overlaid with mean predicted and actual NT-proBNP values with 95% confidence intervals. The alignment between predicted and observed trajectories indicates that the model captures the overall direction of longitudinal changes in NT-proBNP. Mean trajectories are shown with solid (true) and dashed (predicted) lines using the study's context-aware LSTM model.

**Figure 2:**
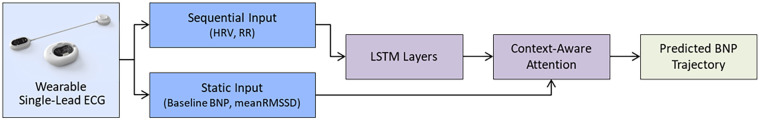
Architecture of the context-aware LSTM model integrating wearable ECG features. The diagram illustrates the full modeling pipeline, combining sequential ECG-derived features (HRV30 min, RR variability, and interaction terms) and static clinical variables (baseline NT-proBNP and meanRMSSD). Three LSTM layers extract temporal patterns, while a context-aware attention mechanism reweights time steps using a clinical context vector. The final dense regression head outputs predicted NT-proBNP trajectories. This architecture enables both dynamic physiologic pattern recognition and patient-specific weighting for predicting NT-proBNP trends.

The best regression performance was achieved when combining clinical metrics, RR-interval features, and long-term HRV summaries (HRV72h), yielding an RMSE of 1,667.04 and an MAE of 950.52. When regression outputs were converted into a trend label (increase vs. decrease/stable), the model achieved directional accuracy of 0.82 (Supplementary Table S1). Predicted and observed NT-proBNP trajectories for individual patients are shown in Figure 1 and Supplementary Table S2. Figure 2 illustrates the architecture of the context-aware LSTM model.

### Key observations

Models using ECG-only features performed comparably to those incorporating multimodal clinical variables.

As shown in Supplementary Table S3, patients with increasing NT-proBNP experienced more HF-related hospitalizations (9 vs. 5), and additional events such as cardioversion and stroke. However, these differences did not reach statistical significance due to the limited number of clinical events.

## Discussion and conclusion

This study provides the first proof-of-concept demonstration that wearable ECG–derived digital biomarkers can predict longitudinal trajectories of NT-proBNP in patients with persistent AF. The comparable performance of ECG-only and multimodal models suggests that RR-interval and autonomic patterns derived from continuous wearable ECG may reflect underlying hemodynamic burden, even in the setting of rhythm irregularity.

Our findings extend prior work that relied on imaging or static clinical parameters for HF risk prediction by demonstrating that non-clinically collected ECG data can yield comparable predictive insights (12, 13). Incorporating such models into telemonitoring workflows may enable earlier identification of worsening physiologic trends and reduce dependence on repeated blood sampling, particularly for elderly AF patients or those with limited access to care.

Several limitations warrant consideration. The study involved a small, single-center cohort without external validation, which limits generalizability. NT-proBNP measurements were obtained at routine visits, resulting in heterogeneous sampling intervals that may introduce temporal noise. Additionally, the NT-proBNP trajectory was used as a surrogate endpoint rather than as a clinical event. Although multiple physiologic factors influence NT-proBNP, its longitudinal trend may more reliably reflect chronic hemodynamic stress than isolated values, especially in persistent AF, where beat-to-beat variability complicates traditional HRV interpretation. Although the fold-wise RMSE variance appeared wide (IQR 1,407, Q1 334.7—Q3 1,741.7), this pattern primarily reflected the large inter-patient heterogeneity in baseline NT-proBNP levels (IQR: 2,145 pg/mL, Q1 821 pg/mL—Q3 2,967 pg/mL), which naturally produces larger absolute errors in patients with high baseline values. This dispersion, therefore, reflects biological variability rather than model overfitting, and the stability of the median fold error indicates that model performance remained robust despite the small sample size. Taken together, these results should be interpreted as exploratory and hypothesis-generating.

Future work will incorporate direction-sensitive loss functions and rolling-slope analysis to enhance the detection of dynamic changes in NT-proBNP, necessitating validation on larger multicenter datasets.

Wearable ECG–based AI modeling shows promise as a scalable, non-invasive approach for monitoring HF progression in persistent AF. With further validation and integration into real-time telemonitoring systems, this strategy may support proactive, personalized management of HF.

## Data Availability

The raw data supporting the conclusions of this article will be made available by the authors upon reasonable request.
