# Supplementary material for: AI-based prediction of heart failure progression in persistent atrial fibrillation using wearable electrocardiography: a brief research report
Source: Front Cardiovasc Med. 2026 Feb 5;12:1748673. doi: 10.3389/fcvm.2025.1748673 (PMC12917893; doi:10.3389/fcvm.2025.1748673)
Supplement: Supplementary file 1 [file Datasheet1.docx]

**Supplementary Methods**

**1. Preprocessing**

(1) R-peak input and noise handling

- R-peak timestamps and beat classifications were provided in inputRrHbClass.csv.
- Noise segments were provided in inputNoise.csv as start-end time pairs.
  - All R-peaks falling with noise-annotated time intervals were removed.
  - If multiple noise intervals existed, removal was performed for each interval.
  - Beat-class labels were not used for ectopic removal, consistent with persistent AF in which beat-to-beat irregularity is physiological.
  - No additional pre-segmentation filtering was applied because raw ECG preprocessing is performed by the device.

(2) Window segmentation

- Each recording was divided into 30-minute windows.
- Within each window, preprocessing proceeded independently.
- If there are fewer than two valid R-peaks remaining after noise removal, the RR interval cannot be calculated, so all HRV values ​​in that window are marked as invalid by assigning -1 (missing code).

(3) RR interval construction and correction

- RR intervals were computed as:

$${RR}_{i}=(t_{i+1}-t_{i})\times1000$$

  - No interpolation or replacement of missing RR intervals was performed at the RR level.
  - Implausible intervals removed earlier via noise exclusion; additionally, windows with too-few RR values were invalidated.

**2. HRV Feature Extraction**

(1) Time-domain HRV feature computation

- Using the cleaned RR series:.
  - SDNN = standard deviation of RR
  - RMSSD = √(mean of squared consecutive RR differences)
  - NN50 = count of $|R_{i}-R_{i+1}|$ > 50 ms
  - pNN50 = NN50 / total RR differences * 100

(2) Frequency-domain HRV feature computation

- Steps per window:
  (a) Convert RR to seconds.
  (b) Compute cumulative timestamps.
  (c) Linearly interpolate RR onto an evenly spaced timeline:

$$t_{interp}=linespace\left( 0, t_{end},N \right)$$

(d) Apply Welch PSD.
  (e) Integrate PSD to obtain:
  - VLF: 0.003 ~ 0.04 Hz
  - LF: 0.04 ~ 0.15 Hz
  - HF: 0.15 ~ 0.40 Hz
  - TP: sum of (VLF, LF, HF)
  - LF/HF: Calculated as LF/HF unless HF = 0

**3. Detailed Model Architecture**

(1) LSTM architecture

- Layer 1: LSTM, 64 units, tahn activation
- Layer 2: LSTM, 32 units, tahn activation
- Layer 3: LSTM, 16 units, tahn activation

(2) Temporal context vector computed using mean pooling over the final LSTM outputs.

(3) Context-aware attention

- The attention mechanism implemented learnable matrices $W$, $U$, bias $b$, and scoring vector v.
  Attention scores were computed as:

$$\alpha_{t}-softmax(v^{\top}\tanh\left( W_{c}+Uh_{t}+b \right))$$

where
  - $h_{t}$: LSTM output at time t
  - $c$: context vector
  - $\alpha_{t}$: normalized temporal attention weight
- The attention-weighted sum of LSTM outputs formed the input to the dense layers.

(4) Regression Head

- Dense: 128, ReLU, L2 = 0.001
- Dropout 0.5
- Dense: 64, ReLU, L2 = 0.001
- Dense: 1 🡪 predicted NT-proBNP change

(5) Training Hyperparameters

- Optimizer: Adam (learning rate 0.001)
- Batch size 8
- Loss: MSE (mean squared error)
- Epochs: 100 maximum
- Early stopping: patience 10

(6) Input Construction

- RR features, HRV72h, HRV30min, and interaction terms formed a multivariate time series
- Clinical metrics were replicated across all time windows
- Final input shape per patient: T X F
- Context-attention additionally received baseline NT-proBNP + meanRMSSD

**4. Statistical Feature Evaluation**

(1) Group-wise comparisons (increase vs. decrease)

- Independent t-tests were conducted for HRV30min features

(2) Linear mixed-effects model

- For each HRV30min features:

$${Feature}_{it}-\beta_{0}+\beta_{1}\left( Time \right)+\beta_{2}\left( Group \right)+\beta_{3}\left( Time\times Group \right)+u_{i}+\epsilon_{it}$$

**5. Linear Regression Slopes**

- Per-patient slopes for HRV30min features were computed:

$${Slope}_{i}=\frac{\Delta{Feature}_{i}}{\Delta t}$$

**6. Feature Prioritization Strategy**

- The following features consistently appeared across all analyses and were therefore assigned higher relevance during model design and interaction-feature construction: maxNN, sdnn, rmssd, tp, lf, hf, lf/hf.
