# Supplementary material for: AI-based prediction of heart failure progression in persistent atrial fibrillation using wearable electrocardiography: a brief research report
Source: Front Cardiovasc Med. 2026 Feb 5;12:1748673. doi: 10.3389/fcvm.2025.1748673 (PMC12917893; doi:10.3389/fcvm.2025.1748673)
Supplement: Supplementary file 4 [file Table3.docx]

| **Clinical Event** | **NT-proBNP Increase (n=21)** | **NT-proBNP Stable/Decrease**  **(n=29)** | **Total (n=50)** |
| --- | --- | --- | --- |
| HF hospitalization | 9 (18%) | 5 (10%) | 14 (28%) |
| AF catheter ablation | 1 (2%) | 4 (8%) | 5 (10%) |
| AF DCCV | 1 (2%) | 2 (8%) | 3 (6%) |
| Stroke | 3 (6%) | 0 (0%) | 3 (6%) |
| **Total (≥1 event)** | 14 (28%) | 11 (22%) | 25 (50%) |

**Supplementary Table 3. Descriptive summary of HF-related clinical events during follow-up, stratified by NT-proBNP trajectory.**

Abbreviations

AF, atrial fibrillation; DCCV, Direct current cardioversion; HF, heart failure; NT-proBNP, N-terminal pro-B-type natriuretic peptide; TIA, transient ischemic attack.
